# Modified Pemberton osteotomy for developmental dysplasia of the hip in children: a mid- to long-term follow-up study

**DOI:** 10.3389/fped.2026.1841163

**Published:** 2026-06-09

**Authors:** Ting Lei, Kun Liu, Jin Tang, Jiang-yan Wu, Qian Tan, Wei-hua Ye, Guang-hui Zhu, Yu-qing Li

**Affiliations:** 1 Orthopedic Department, Hunan Children’s Hospital (The Affiliated Children’s Hospital of Xiangya School of Medicine, Central South University), Changsha, Hunan, China; 2 The School of Pediatrics, University of South China, Changsha, Hunan, China; 3 Hunan Provincial Key Laboratory of Pediatric Orthopedics, Changsha, Hunan, China

**Keywords:** acetabular index, avascular necrosis, hip dysplasia, modified pemberton osteotomy, pediatric pelvic osteotomy

## Abstract

**Background and objective:**

Developmental dysplasia of the hip (DDH) is a common pediatric condition requiring timely surgical intervention in older children. Conventional Pemberton osteotomy, while effective, carries a risk of iatrogenic injury to the triradiate cartilage due to its blind deep-pericapsular trajectory. The modified Pemberton osteotomy (MPO) shifts the osteotomy endpoint to 3–5 mm above the sciatic notch, creating a greenstick hinge above the Y-shaped cartilage to avoid growth-plate damage. This study aimed to evaluate the mid- to long-term efficacy and safety of MPO in children with DDH over a minimum follow-up of 5 years.

**Materials and methods:**

We retrospectively reviewed 55 patients (71 hips) who underwent open reduction combined with MPO between May 2004 and June 2025. Radiographic parameters included acetabular index (AI), central-edge angle (CEA), and Reimer index (RI). Clinical and radiographic outcomes were assessed using McKay criteria and Severin classification, respectively.

**Results:**

The mean follow-up duration was 7.21±1.64 years. The mean AI improved from 39.66 ± 5.46° preoperatively to 12.71 ± 8.37° at final follow-up, demonstrating powerful and stable acetabular remodeling capacity. According to the McKay clinical criteria, highly satisfactory functional outcomes were achieved, with an excellent-good rate of 88.7% (63/71 hips). Radiographic assessment using the Severin classification confirmed excellent joint congruence, yielding an excellent-good rate of 93.0% (66/71 hips). Avascular necrosis (AVN) occurred in 8 hips (11.2%), which is a low and acceptable incidence compared to historical pelvic osteotomies.

**Conclusion:**

MPO provides favorable and durable mid- to long-term clinical and radiographic outcomes for DDH in children. By avoiding iatrogenic injury to the triradiate cartilage, MPO offers improved surgical safety compared with conventional Pemberton osteotomy, with an acceptable AVN rate directly attributable to the procedure.

## 1 Introduction

Developmental dysplasia of the hip (DDH) is a common pediatric condition that may be present at birth or develop progressively with age, characterized by loss of normal anatomical congruence between the femoral head and acetabulum leading to hip subluxation or dislocation, with early diagnosis and intervention being crucial for optimal outcomes (1). Treatment strategies vary by age: children younger than 6 months are typically treated with Pavlik harness or bracing (2). Those aged 6–18 months may receive closed or open reduction with cast immobilization, while pelvic or combined femoral osteotomy is generally indicated for children older than 2 years, with Salter and Pemberton being the most commonly used pelvic osteotomies (3). Perlik first reported a combined osteotomy in 1985, describing an osteotomy line crossing the posterior Y-shaped cartilage and extending to the sciatic notch (4). However, conventional Pemberton osteotomy poses a subtle risk of iatrogenic injury to the triradiate cartilage due to its blind, deep pericapsular trajectory (5). To address this limitation, our institution adopted the modified Pemberton osteotomy (MPO). The primary motivation behind this technical modification is to improve safety through direct visualization and cartilage preservation. By shifting the osteotomy trajectory superiorly to terminate 3–5 mm above the sciatic notch, MPO creates a rotational hinge above the triradiate cartilage, minimizing growth-plate disturbance (6). Concurrently, it permits full visualization of the sciatic notch margins, structurally reducing surgical difficulty.

This study retrospectively reviewed DDH children treated with MPO at our hospital from May 2004 to June 2025 (minimum 5-year follow-up), evaluating radiographic parameters including correction of acetabular index (AI), final AI, and central-edge angle (CEA), reimer index (RI), along with functional outcomes assessed by Severin x-ray grading and McKay clinical hip function scores. The surgical outcomes demonstrated MPO's effectiveness in restoring hip joint anatomy and function, with detailed results presented in subsequent sections.

## 2 Materials and methods

### 2.1 Patient selection and baseline characteristics

**Figure 1:**
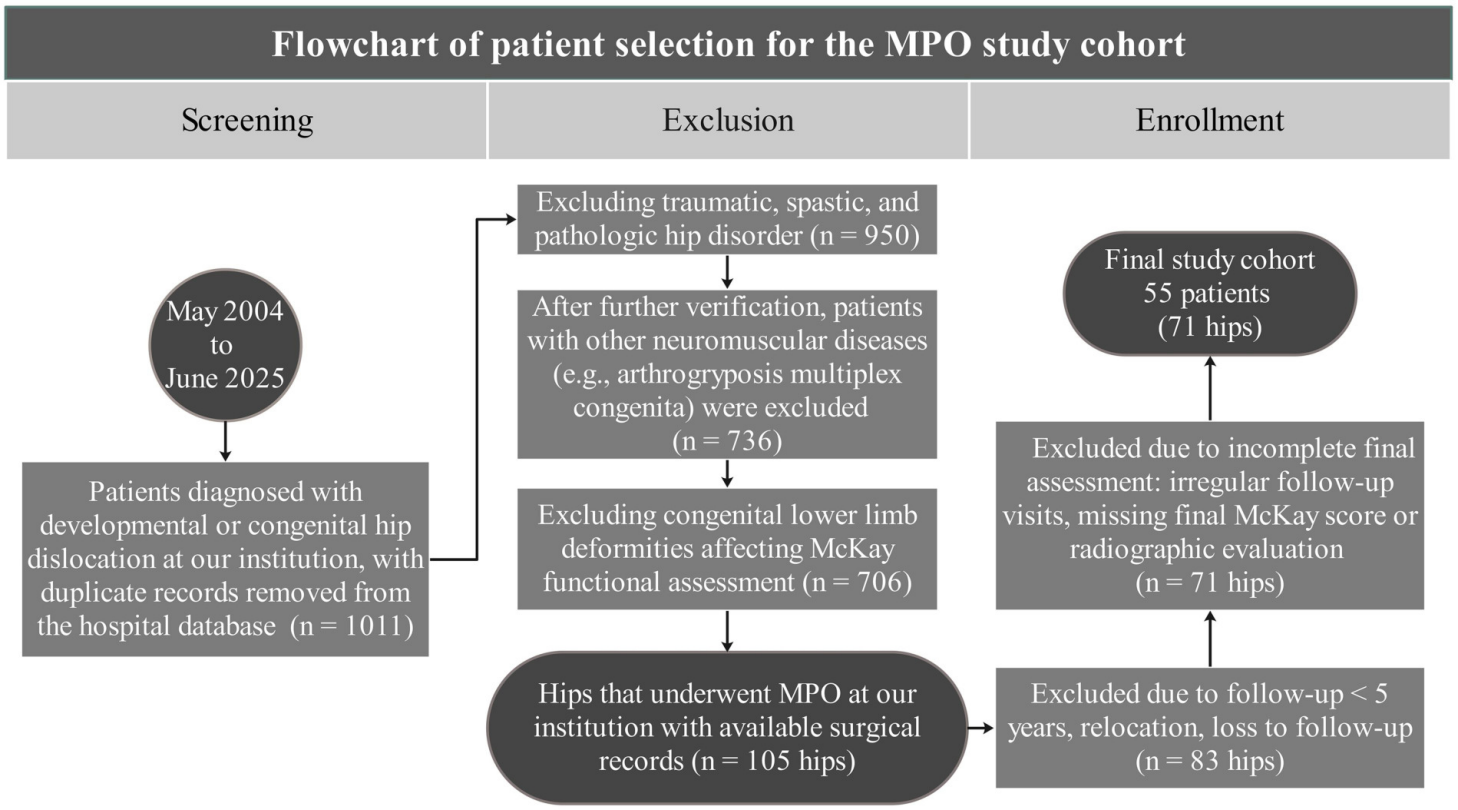
Patient screening, exclusion, and final enrollment process for the MPO study cohort. MPO: modified Pemberton osteotomy; “n” denotes the number of records (patients or hips) remaining after each step. A total of 55 patients (71 hips) were ultimately included. Mean follow-up: 7.21 ± 1.64 years.

This retrospective study included children with DDH who underwent MPO at our institution between May 2004 and June 2025. Inclusion criteria were: (1) diagnosis of DDH; (2) treatment with MPO; (3) minimum follow-up of 5 years. Exclusion criteria were: (1) traumatic, spastic, or pathologic hip disorders; (2) other neuromuscular diseases (e.g., arthrogryposis multiplex congenita); (3) congenital lower limb deformities that could affect McKay functional assessment; (4) incomplete medical records or loss to follow-up. The patient selection process is summarized in Figure 1.

After applying these criteria, 55 children (71 hips) were included. The cohort comprised 11 males (14 hips) and 44 females (57 hips), with a mean age of 3.62 ± 1.78 years at surgery. For bilateral cases (*n* = 16), the contralateral procedure was performed 6–12 months after the initial operation. The mean follow-up duration was 7.21±1.64 years (range: 4.7–10.4 years). One patient with bilateral DDH underwent staged MPO. One hip was followed for 5.3 years, while the contralateral hip had a follow-up of only 4.7 years (less than the required 5 years). This patient received a poor McKay rating. Excluding this case would have substantially affected the study results; therefore, it was retained in the analysis. Preoperative evaluation revealed a mean acetabular index (AI) of 39.66 ± 5.46°, with Tonnis classification grade II (19 hips), III (40 hips), and IV (12 hips) (7). Detailed baseline characteristics are presented in Table 1.

**Table 1:** Baseline characteristics of 55 patients (71 hips) with DDH undergoing MPO.

Characteristic	Age at surgery (years)	Follow-up duration (years)	Sex, *n* (%)	Laterality (patients), *n* (%)	Surgical details, *n* (%)	Pre-Tönnis classification, *n* (%)
Value	3.62 ± 1.78	7.21 ± 1.64	Male: 11 (20.0) Female: 44 (80.0)	Unilateral 39 (70.9%) Bilateral 16 (29.1%)	Concomitant femoral osteotomy: 67 (94.4) Isolated pelvic osteotomy: 4 (5.6)	Grade II: 19 (26.8) Grade III: 40 (56.3) Grade IV: 12 (16.9)

Pre, Preoperative; Data are presented as mean ± standard deviation (SD), *n* (%), or raw counts.

### 2.2 Surgical technique

All procedures included open reduction and pelvic osteotomy with autologous or allogeneic bone grafting. Femoral osteotomy was performed in 52 cases (67 hips), while 3 cases (4 hips) underwent isolated pelvic procedures. The surgical approach utilized a standard bikini incision, with an additional lateral longitudinal incision for cases requiring femoral shortening. Key anatomical landmarks [anterior superior iliac spine (ASIS), anterior inferior iliac spine (AIIS) and sciatic notch] were thoroughly exposed to achieve complete visualization of the medial and lateral margins of the notch, thereby reducing the technical difficulty and ensuring precise osteotomy placement.

**Figure 2:**
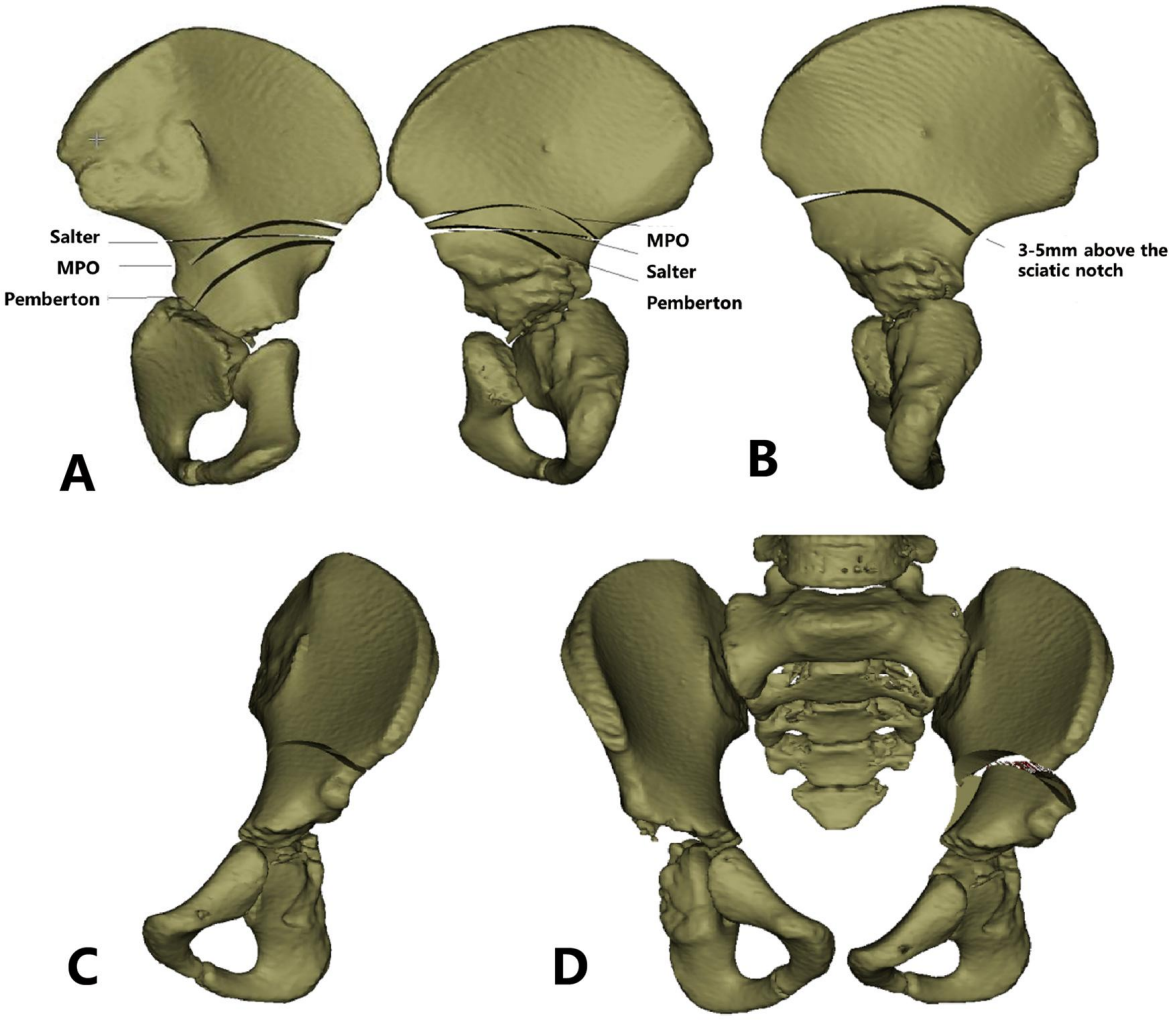
Changes in acetabular volume and obturator foramen following iliac bone block rotation via the MPO osteotomy line. **(A)** Schematic comparison of osteotomy lines in MPO, Salter, and Pemberton procedures **(B)** The MPO osteotomy line is positioned 3–5 mm above the sciatic notch. **(C)** Complete osteotomy line in MPO. **(D)** Osteotomy distraction and valgus correction.

Outer table osteotomy (Figures 2A,B): A curvilinear incision was made parallel to the posterior acetabular margin, extending from the midpoint between the ASIS and AIIS to 3–5 mm superior to the sciatic notch. Unlike the classical Pemberton procedure, the MPO trajectory is intentionally shifted superiorly to terminate above the sciatic notch rather than deep within the ilium. This design modification is primarily motivated by the need to establish a rotational hinge via a controlled greenstick fracture above the triradiate cartilage, thereby effectively shielding the “Y-shaped” growth plate from inadvertent iatrogenic injury. Inner table osteotomy: Beginning at the same starting point, a parallel arc was created but terminated proximal to the sciatic notch. By avoiding a blind, deep pericapsular cut and ensuring the osteotomy remains within a visible range, this approach converts the procedure into a more controlled and maneuverable intervention, significantly reducing the risk of articular or growth plate damage.

**Figure 3:**
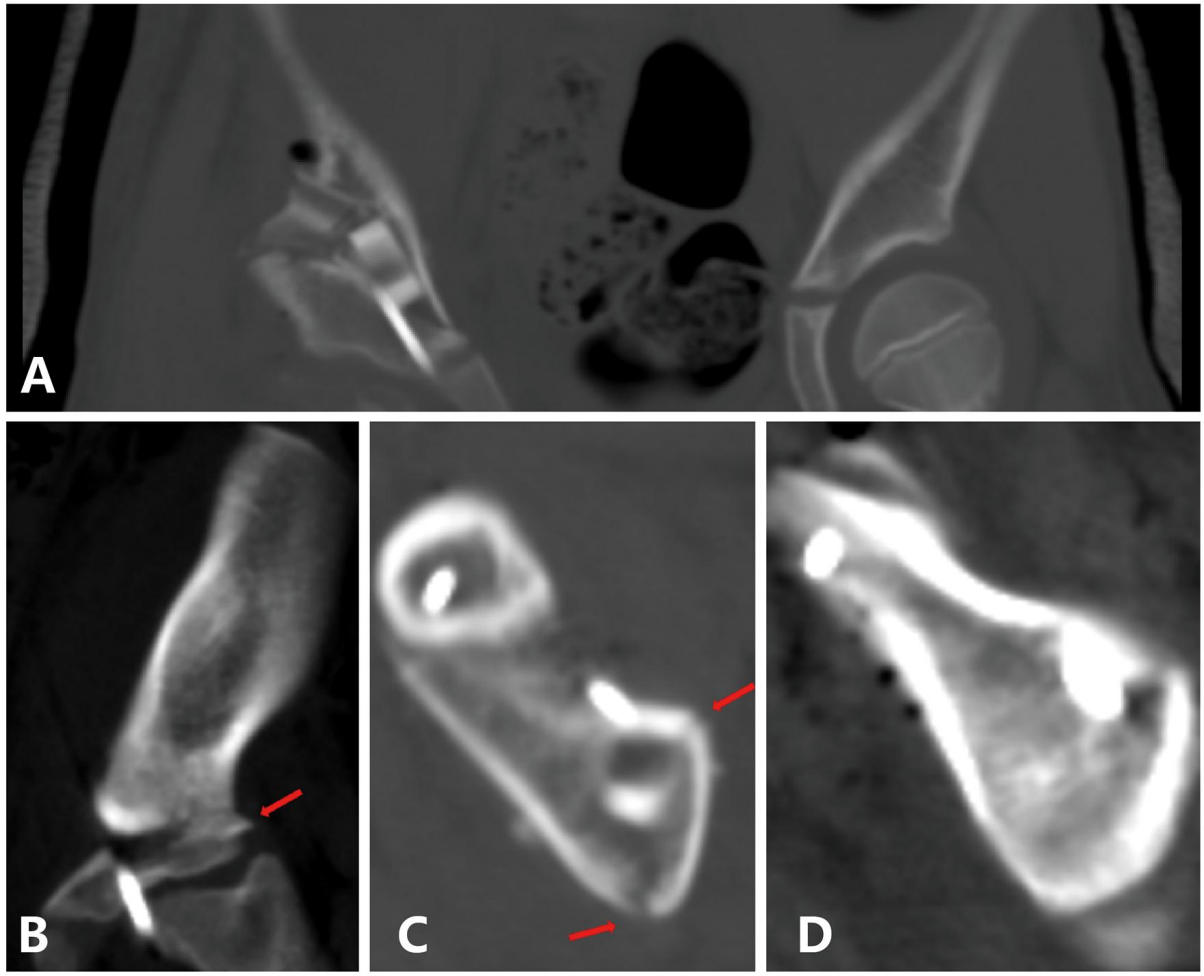
Distraction and fixation following an MPO osteotomy. **(A)** Postoperative CT scan of right DDH showing outward rotation of the osteotomy fragment. **(B)** Sagittal CT View. The red arrow indicates a greenstick fracture at the sciatic notch. The Y-shaped cartilage and superior bone structures remain intact. **(C)** Axial CT View at Osteotomy Level. The inferior arrow marks the terminus of the external iliac plate osteotomy, which serves as the center of rotation, with outward rotation of the distal segment. The superior red arrow indicates opening of the internal iliac plate. The femoral autograft is fixed within this gap, positioned closer to the sciatic notch compared to the conventional periacetabular osteotomy. **(D)** Axial CT View Below Osteotomy Level. At this adjacent inferior level, the iliac cortex is continuous, and the osteotomy line is no longer visible.

**Figure 4:**
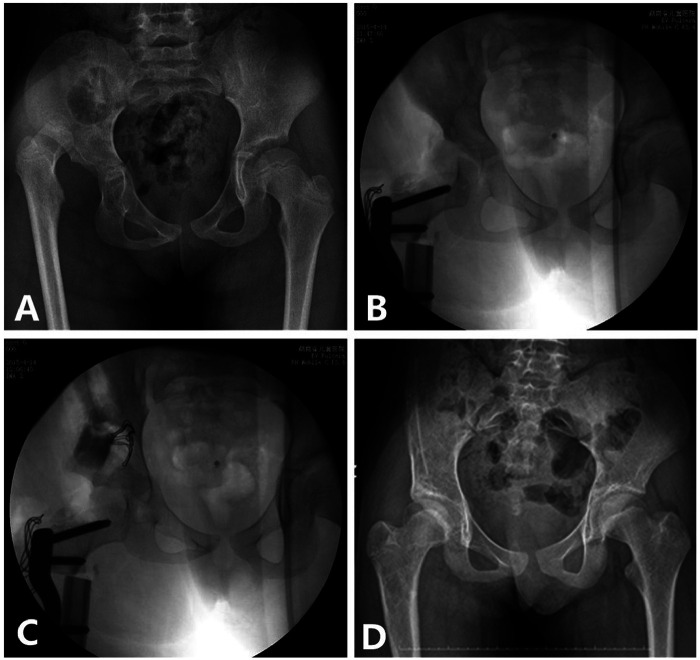
Representative case of MPO for right DDH. **(A)** Preoperative anteroposterior pelvic radiograph (8y3 m, F) showing hip dislocation and increased AI. **(B)** Intraoperative fluoroscopy confirming central reduction. **(C)** Immediate postoperative radiograph demonstrating improved coverage. **(D)** 6-year follow-up showing maintained reduction, remodeled acetabulum, and no AVN signs.

Osteotomy distraction and fixation (Figures 2C,D; Figures 3, 4): Following osteotomy completion, a lamina spreader was carefully inserted into the osteotomy gap to achieve controlled distraction of the lateral acetabular fragment. This maneuver created a controlled cortical breach at the osteotomy terminus, establishing a rotational fulcrum that enabled precise outward displacement of the acetabular segment—a mechanism biomimetically replicating the Pemberton osteotomy principle. Simultaneously, a secondary controlled fracture at the sciatic notch produced a partially mobile yet structurally continuous pelvic ring, analogous to the pubic symphysis-centered rotation mechanics of Salter osteotomy. Upon achieving satisfactory acetabular reorientation (AI < 20°), the distraction gap was reconstructed using precisely contoured autologous iliac crest grafts, femoral shortening segments, or allogeneic bone blocks, which provided sufficient structural support to maintain correction without supplemental internal fixation in most cases.

### 2.3 Postoperative treatment

Immediately after surgery, a pelvic radiograph was obtained to confirm concentric reduction of the hip and an AI of less than 20° (Figure 4C). A hip spica cast was then applied with the hip positioned at 20° of flexion and maintained in abduction. The cast was maintained for 6–8 weeks. After cast removal, partial weight-bearing walking was initiated under the supervision of a physical therapist, progressing to full weight-bearing according to radiographic evidence of bone healing and functional recovery. For patients with bilateral DDH who required a second-stage contralateral MPO, the second operation was performed only after the first hip had achieved full, painless range of motion, typically 6–12 months after the initial procedure. Internal fixation (if used) was removed approximately one year postoperatively after confirmation of complete bone union.

### 2.4 Follow up and evaluation criteria

Follow-up Protocol: Clinical and radiographic evaluations were conducted at 3 months and >5 years postoperatively. The final follow-up timepoint was defined as: (1) the last available follow-up for non-revision cases (Figure 4D); (2) if a second revision operation was performed, the time of this second revision operation served as the final follow-up time. For AI measurement, when the final follow-up AI was unmeasurable due to triradiate cartilage fusion, the Sharp angle was substituted as the standard radiographic measurement for acetabular dysplasia in such patients. AI, CEA, and RI were measured at preoperative, postoperative, and final follow-up. The excellent/good rates based on Severin radiographic classification and McKay hip clinical function were evaluated at final follow-up (8, 9). Avascular necrosis of the femoral head was evaluated at final follow-up by Kalamchi & MacEwen (10).

## 3 Result

As shown in Table 2, the mean acetabular index (AI) improved significantly from 39.66 ± 5.46° preoperatively to 12.71 ± 8.37° at final follow-up (*P* < 0.05). McKay hip function evaluation demonstrated excellent results in 48 hips (67.6%), good in 15 hips (21.1%), fair in 7 hips (9.9%), and poor in 1 hip (1.4%), yielding an 88.7% excellent-good rate. The single poor outcome occurred in a bilateral DDH case that developed right femoral fracture 6 months postoperatively, progressing to bilateral type IV AVN (Kalamchi & MacEwen classification) with right hip contracture and persistent pain. Despite surgical release and continuous passive motion therapy, right hip mobility remained limited to 0–30° flexion-extension (vs. 0–90° on the contralateral pain-free side), resulting in a final fair rating.

**Table 2:** Radiographic parameters (AI, CEA, RI) at preoperative, postoperative, and final follow-up assessments.

Parameter	AI°	RI (%)	CEA°	Severin E/G rate	Mckay E/G rate
Preoperative	39.66 ± 5.46	-	-	-	-
Final follow up	12.71 ± 8.37*	12.00 ± 10.10	34.85 ± 6.48	93.0%	88.7%

AI, acetabular index correction; CEA, central-edge angle; RI, reimer index; E/G, excellent/good rates based on Severin radiographic classification and McKay clinical hip function evaluation; Paired samples *t*-test, preoperative vs. final follow-up; *P* < 0.05. Values are mean ± SD (95% CI). CEA and RI could not be measured preoperatively due to hip dislocation and are therefore reported only at final follow-up. n = 71 hips for all analyses. *P*<0.05 compared with the preoperative value (indicated by *).

Severin radiographic assessment showed excellent results in 51 hips (71.8%), good in 15 hips (21.1%), fair in 2 hips (2.8%), and poor in 3 hips (4.2%), achieving a 93.0% excellent-good rate. Avascular necrosis (AVN) distribution was: grade I in 2 hips (2.8%), grade II in 1 hip (1.4%), grade III in 2 hips (2.8%), and grade IV in 3 hips (4.2%), with an overall AVN incidence of 11.2%. Femoral head height restoration occurred in 4 hips (5.6%), while 4 hips (5.6%) developed permanent deformity.

Additional complications included: (1) Two cases (2.8%) of proximal femoral fracture secondary to low-energy trauma, both treated with open reduction and LCP fixation, achieving excellent McKay/good Severin outcomes; (2) Five unilateral cases requiring epiphyseal block for limb length discrepancy (mean 2.7 cm, range 2.2–3.9 cm; 4 affected side longer, 1 contralateral longer), all achieving equal limb length at final follow-up.

## 4 Statistical analysis

Statistical analyses were performed using SPSS 26.0 (IBM Corp., Armonk, NY, USA). The primary statistical comparison was a paired samples t-test comparing preoperative and final follow-up acetabular index (AI) within the study cohort; results are presented as mean ± standard deviation (SD) with 95% confidence intervals (95% CI). To assess potential selection bias, baseline characteristics (age, sex, preoperative AI) of excluded hips were compared with the included cohort using independent samples t-tests and chi-square tests. A two-tailed *P*-value < 0.05 was considered statistically significant.

## 5 Discussion

In this study, modified Pemberton osteotomy (MPO) demonstrated favorable efficacy in correcting the acetabular index (AI), with a mean postoperative AI of 26.63° ± 7.35°. Postoperative radiographs in some cases presented challenges in identifying the acetabular sourcil; therefore, the lateral edge of the acetabulum was used as the landmark for AI measurement, which may introduce a slight measurement bias. Kothari compared measurements taken at the lateral edge vs. the sourcil, reporting a statistically significant mean difference of 2.42° between the two methods (11). Grafting techniques included allogeneic iliac bone or femoral autografts to address residual gaps. Bulut observed no significant difference in final AI between these grafting methods (12).

Early postoperative imaging indicated nearly complete femoral head coverage, likely attributable to the more superior starting point of the osteotomy compared to conventional Pemberton or Salter techniques. However, over extended follow-up, absorption and remodeling of the lateral acetabular cartilage were observed, leading to a gradual decline in CE angle and an increase in RI. Li suggested that the RI may be less reliable than AI for assessing acetabular development (13). Avascular necrosis (AVN) of the femoral head occurred in 8 hips (11.2%), 4 of which showed radiographic improvement at final follow-up. One patient (2 hips) developed severe type IV AVN with coxa vara, short neck deformity, and joint stiffness. Reported AVN rates in the literature vary widely (0%–60%) (14–16). Our AVN rate of 11.2% represents complications directly related to MPO, as patients with pre-existing AVN from prior closed reduction were excluded. This distinction may partly explain the comparatively lower incidence in our series. Functional outcomes assessed via McKay criteria and Severin classification were generally favorable. These findings are highly consistent with the successful therapeutic benchmarks established in the recent comprehensive review by Sang regarding the surgical management of residual and late-presenting acetabular dysplasia (17). They emphasized that the primary goals of pelvic osteotomy are to optimize femoral head coverage and delay osteoarthritis, while minimizing iatrogenic complications. Our clinical success rate (McKay excellent-good rate of 88.7%) and radiographic remodeling (Severin excellent-good rate of 93.0%) match the top-tier outcomes compiled in their review. Furthermore, avascular necrosis remains a major concern in rigid open reductions and redirectional osteotomies. Our overall AVN rate of 11.2% is not only at the lower end of the traditional 0%–60% range, but also confirms that MPO's safety profile is excellent, as it completely protects the triradiate cartilage from iatrogenic damage via its superior greenstick hinge design.

Pelvic osteotomies represent established interventions for developmental dysplasia of the hip (DDH), operating primarily through two distinct biomechanical mechanisms: reorientation of the acetabulum (e.g., Salter osteotomy) or alteration of its structural depth (e.g., Pemberton osteotomy). Salter osteotomy, which entails a complete transiliac osteotomy rotated about the pubic symphysis, enhances anterior and lateral femoral head coverage by redirection of the acetabulum while preserving its native volume (18). This technique is ideally indicated in patients with satisfactory congruity between the femoral head and acetabulum, although its capacity for AI correction is typically limited to approximately 15°. Long-term follow-up studies indicate comparable outcomes between Salter and Pemberton osteotomies regarding both AI and CE angle (19, 20). In contrast, Pemberton osteotomy utilizes an incomplete pericapsular osteotomy hinged on the triradiate cartilage, effectively deepening the acetabulum while reducing its volume (21). This approach confers greater improvement in acetabular depth compared to the Salter technique and can be performed bilaterally in a single stage due to maintenance of pelvic ring integrity (20). However, it necessitates meticulous exposure of the triradiate cartilage and entails a risk of iatrogenic acetabular injury (22).

To mitigate the risk of iatrogenic acetabular injury, our MPO technique incorporates a modified osteotomy trajectory, shifting the starting point superiorly toward the midpoint between the anterior superior and inferior iliac spines and ending 3–5 mm above the sciatic notch. The inner and outer cortical cuts follow parallel arcs, with the inner cut terminating anterior to the sciatic notch. This modification facilitates surgical exposure and reduces the risk of cartilage injury (Figure 2). During distal segment rotation, a greenstick fracture occurs near the sciatic notch, creating a hinge effect analogous to that in Pemberton osteotomy, while the broader osteotomy base and more posterior hinge also introduce a reorienting effect similar to Salter osteotomy (Figure 3). Greater AI correction was associated with more pronounced obturator foramen deformation, reflecting increased Salter-like rotation. In our series, no iatrogenic acetabular injuries occurred among the 55 patients. But we had a high loss to follow-up, particularly beyond 1–2 years postoperatively, precluding longitudinal regression analysis of AI, CE angle, and RI evolution.

In this study, 34 hips were excluded due to follow-up <5 years or incomplete final assessment. To assess potential selection bias, we compared baseline characteristics between the excluded hips and the included cohort (71 hips). No significant differences were observed in age, sex, or preoperative acetabular index (AI) (*p* > 0.05 for all). However, postoperative follow-up data were not available for the excluded hips, and their clinical outcomes remain unknown. Therefore, although baseline comparability reduces the likelihood of severe selection bias, we cannot completely rule out the possibility that the excluded group might have had systematically different outcomes (e.g., poorer results leading to loss of follow-up). This inherent limitation of retrospective studies should be considered when interpreting our excellent-good rates. As this is a single-center study, the reproducibility of MPO across different institutions remains to be validated by future multicenter case series.

While Pemberton osteotomy is traditionally recommended for children under 6 years of age, satisfactory outcomes with MPO have been observed in older children as well (23). In our cohort, 10 hips were in children over 6 years old, with excellent or good McKay results achieved in 70% of these cases. Key technical considerations of MPO include: (1) thorough exposure of the anterior iliac spines and sciatic notch with pre-osteotomy marking; (2) preserving sciatic notch cortical continuity, intentionally inducing a greenstick fracture during rotation rather than performing a complete osteotomy; (3) adequately widening the osteotomy gap based on preoperative AI to avoid impingement; and (4) employing Salter-like fixation if complete sciatic notch fracture occurs to prevent persistent separation or instability.

## 6 Conclusion

In conclusion, the MPO provides a reliable and safe option for treating DDH in children. By shifting the osteotomy terminus toward the sciatic notch and creating a hinge above the triradiate cartilage, MPO achieves satisfactory acetabular remodeling and mid- to long-term clinical outcomes while minimizing the risk of iatrogenic growth plate injury.

## Data Availability

The original contributions presented in the study are included in the article/Supplementary Material, further inquiries can be directed to the corresponding authors.

## 7 Glossary

Acetabular index (AI): the angle formed by the horizontal reference line (Hilgenreiner line) and a line through the lateral edge of the acetabular sourcil; it is measured only when the triradiate cartilage remains open, and values exceeding 30° indicate acetabular dysplasia.
Central-edge angle (CEA): the angle between a vertical line through the center of the femoral head and a line connecting this center to the lateral acetabular margin; a CEA greater than 25° in adolescents or greater than 19° in children aged 6–13 years is considered normal.
Sharp angle (acetabular angle): the angle formed by the horizontal reference line (Hilgenreiner line) and a line from the caudal tip of the teardrop to the lateral edge of the acetabulum; used after fusion of the triradiate cartilage, with normal values <32° and values >42° indicating dysplasia.
Reimer index (RI): the percentage of the femoral head that remains uncovered lateral to the acetabular edge relative to the total femoral head diameter; a value below 33% is normal.
Severin classification: grades radiographic outcome from grade I (excellent) to grade VI (poor), with grades I–II regarded as satisfactory (excellent/good).
McKay clinical criteria: evaluates hip function based on gait, range of motion, pain, and limp; excellent or good results are considered satisfactory.
